# Identification of Potential Dipeptidyl Peptidase (DPP)-IV Inhibitors among *Moringa oleifera* Phytochemicals by Virtual Screening, Molecular Docking Analysis, ADME/T-Based Prediction, and In Vitro Analyses

**DOI:** 10.3390/molecules25010189

**Published:** 2020-01-02

**Authors:** Yang Yang, Chong-Yin Shi, Jing Xie, Jia-He Dai, Shui-Lian He, Yang Tian

**Affiliations:** 1 College of Science, Yunnan Agricultural University, Kunming 650201, China; 2 Yunnan Key Laboratory of Biomass Big Data, Yunnan Agricultural University, Kunming 650201, China; jingxie0624@163.com; 3 Institute of Food Science and Technology, Yunnan Agricultural University, Kunming 650201, China; shichongying@163.com (C.-Y.S.); keloyy@163.com (J.-H.D.); 4 College of Horticulture and Landscape, Yunnan Agricultural University, Kunming 650201, China

**Keywords:** *Moringa oleifera*, dipeptidyl peptidase IV, type 2 diabetes, virtual screening, molecular docking

## Abstract

*Moringa oleifera* Lam. (MO) is called the “Miracle Tree” because of its extensive pharmacological activity. In addition to being an important food, it has also been used for a long time in traditional medicine in Asia for the treatment of chronic diseases such as diabetes and obesity. In this study, by constructing a library of MO phytochemical structures and using Discovery Studio software, compounds were subjected to virtual screening and molecular docking experiments related to their inhibition of dipeptidyl peptidase (DPP-IV), an important target for the treatment of type 2 diabetes. After the four-step screening process, involving screening for drug-like compounds, predicting the absorption, distribution, metabolism, excretion, and toxicity (ADME/T) of pharmacokinetic properties, LibDock heatmap matching analysis, and CDOCKER molecular docking analysis, three MO components that were candidate DPP-IV inhibitors were identified and their docking modes were analyzed. In vitro activity verification showed that all three MO components had certain DPP-IV inhibitory activities, of which O-Ethyl-4-[(α-l-rhamnosyloxy)-benzyl] carbamate (compound **1**) had the highest activity (half-maximal inhibitory concentration [IC_50_] = 798 nM). This study provides a reference for exploring the molecular mechanisms underlying the anti-diabetic activity of MO. The obtained DPP-IV inhibitors could be used for structural optimization and in-depth in vivo evaluation.

## 1. Introduction

Diabetes mellitus (DM) is a metabolic disorder characterized by long-term high blood glucose levels [1]. Over time, DM can severely damage the heart, kidneys, and nervous system. DM is divided into two categories (types 1 and 2) based on an absolute or relative lack of insulin among patients. Patients with type 2 DM account for about 90% of patients with DM. Recent data from the World Health Organization (WHO) indicate that DM affected >422 million people worldwide (8.5% of the global population) in 2014, which may increase to 592 million by 2035. In 2016, DM ranked seventh among medical conditions in terms of its mortality rate, and it has become one of the major threats to human health worldwide [2].

Glucagon-like peptide (GLP)-1 is an insulin-promoting polypeptide produced by intestinal endocrine cells. The intake of sugars and lipids stimulates the release of GLP-1 [2]. GLP-1 receptor (GLP-1R) is a G protein-coupled receptor (GPCR). GLP-1 binds to GLP-1R and activates adenylyl cyclase to generate cAMP. The protein kinase A (PKA) and exchange protein directly activated by cAMP (EPAC2) dual targets enhance glucose-stimulated insulin secretion [3]. GLP-1′s hypoglycemic mechanism is safe because GLP-1 promotes insulin secretion in a glucose concentration-dependent manner. GLP-1 stimulates increased insulin secretion when the blood glucose level rises, but when the blood glucose level is too low it appears to maintain normal insulin secretion levels [4].

Along with GLP-1, incretins (a group of metabolic hormones that promote insulin secretion) also include glucose-dependent insulinotropic polypeptide (GIP), which plays a similar role in stimulating insulin secretion. However, both peptides are very unstable in vivo, with extremely short half-lives; they are easily degraded and inactivated by dipeptidyl peptidase (DPP)-IV enzymes [5]. DPP-IV, also called CD26, is a serine protease. Prolonging the hypoglycemic effects of GLP-1 and GIP by inhibiting DPP-IV is one of the key mechanisms of type 2 DM treatment [6]. DPP-IV inhibitors currently on the market include sitagliptin and vitagliptin, which have significant hypoglycemic effects. However, they have certain side effects such as hypersensitivity reactions, rashes, and upper respiratory tract infections [7]. Therefore, the discovery of DPP-IV inhibitors with new structures, especially among the secondary metabolites of plants, with higher safety, is a reliable and proven approach for discovering new hypoglycemic drugs [8]. For example, Gao et al. conducted a review and reported that 63 kinds of natural flavonoids and alkaloids that inhibit DPP-IV have been found, which proves that natural products are an important source of active drugs [9].

Nowadays, it takes 10–15 years to develop a new drug and costs more than $800 million [10]. Due to lack of certain drug-like properties, poor pharmacokinetic properties, and toxicity problems that arise in clinical trials, only one drug among 10,000 candidates may eventually come to market during the drug development process [11]. With the development of computer technology, structure-based virtual screening has become one of the core technical methods for drug discovery [12]. Using the high-throughput method of virtual screening, the properties of candidates can be evaluated, such as by using Lipinski’s “rule of five” and predicting human intestinal absorption (HIA), blood–brain barrier (BBB) penetration, cytochrome P450 2D6 (CYP2D6) inhibition, plasma protein binding, aqueous solubility, and toxicity [13]. Thus, candidate compounds that have low drugability can be excluded, decreasing costs and increasing efficiency [14].

Molecular docking analysis is a drug discovery technology based on the simulation of interactions between a ligand and target protein (including GPCRs, ion channels, protein kinases, and nuclear hormone receptors). Libraries of compound structures from different sources have been screened and analyzed by pharmacophore matching and molecular docking methods, based on the drug-binding sites of the target proteins [15]. Virtual drug screening based on molecular docking technology has become a popular and effective drug development strategy. For example, Berin et al. discovered flavonoid molecules with sirtuin protein inhibitory activity in the African natural product library [16], and Faraz et al. identified Ebola virus inhibitors in traditional Chinese medicine databases [17], thereby using efficient virtual screening methods to discover new active compounds with clear potential molecular mechanisms. 

*Moringa oleifera* Lam. (MO) has been called the “Tree of Life” and “Miracle Tree” in tropical and subtropical regions for a long time, due to it being an important food and a traditional medicine in Asia for treating diabetes and obesity [18]. At present, there are few studies on the pharmacological activity of the chemical constituents of MO, and these studies have been limited to the exploration of the apparent bioactivity of crude extracts. For example, Perumal et al. found that MO extracts have antihypertensive and hypoglycemic activity [19]. Jorge et al. found that MO leaf extract exerts hypoglycemic activity by regulating mitochondrial respiration [20]. Most studies on the hypoglycemic activity of MO extracts have not identified an individual component with a specific molecular mechanism. 

In this study, a virtual library of MO phytochemicals was established, and potential DPP-IV inhibitors were discovered by virtual screening based on drug-like properties and molecular docking evaluation principles, and the inhibition of DPP-IV was confirmed by in vitro experiments. Three potential DPP-IV inhibitors of MO origin were discovered for the first time, and the study revealed the possible anti-diabetic molecular mechanism. The three DPP-IV inhibitors could be used as the basis for further structural optimization and in vivo research. 

## 2. Results and Discussion

A virtual library of 111 compounds that isolated from MO was established using a database search (Table S1 in Supplementary Materials). First, based on Lipinski’s “rule of five”, molecules with less reasonable physicochemical properties were discarded [21], leading to the selection of 64 candidate molecules with good drug-like properties: molecular weight < 500, number of hydrogen bond donors < 5, number of hydrogen bond acceptors < 10, ALogP < 5, and no more than one violation of the above criteria.

**Figure 1:**
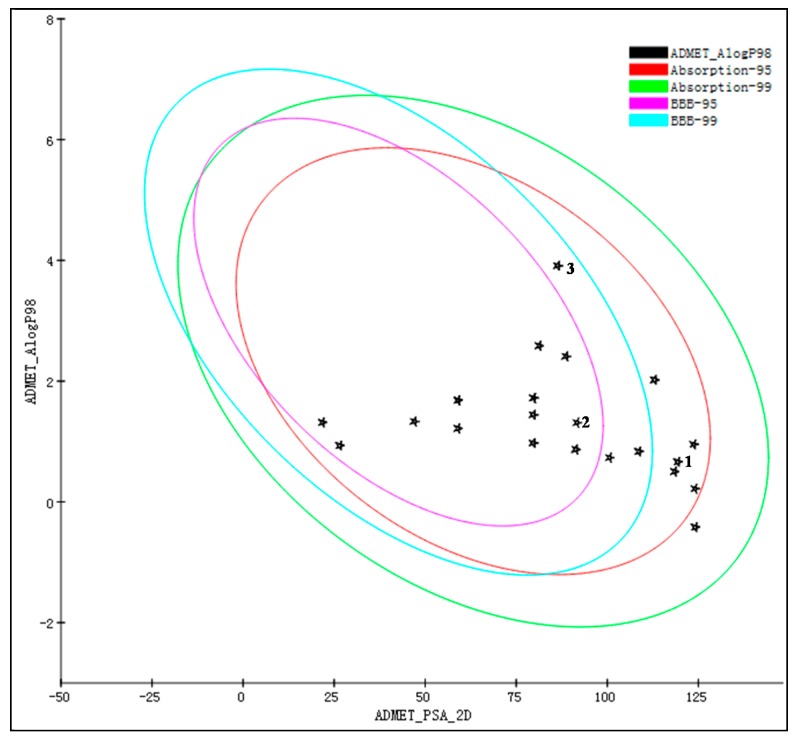
Relationship between the two-dimensional polar surface area (PSA_2D) and the calculated value of AlogP98 of 23 candidate compounds selected after absorption, distribution, metabolism, excretion, and toxicity (ADME/T) screening, showing the corresponding blood–brain barrier (BBB) penetration and human intestinal absorption (HIA) model 95% and 99% confidence ellipses.

Next, the “ADME/T descriptors” (absorption, distribution, metabolism, excretion, and toxicity) and “toxicity prediction” modules were used to predict the pharmacokinetic and toxicity parameters of the 64 candidate molecules. We excluded molecules that are difficult for the intestine to absorb, easily penetrate the BBB, inhibit CYP2D6, have a high plasma protein binding rate, have poor water solubility, and are toxic (high probability of carcinogenicity and mutagenesis), leaving 23 candidate compounds [22]. The relationship between the two-dimensional polar surface area (PSA_2D) and the calculated value of AlogP98 for the 23 compounds is shown in Figure 1, with the HIA and BBB penetration model 95% and 99% confidence ellipses. Predicting the value of AlogP98 can determine the hydrophilicity of the compound. AlogP98 > 5 may be related to the absorption or permeability of the compound. PSA is another key attribute related to drug bioavailability, as compounds with PSA <140 Å^2^ can be passively absorbed and so have high oral bioavailability [23]. As shown in Figure 1, the 23 compounds all fell within these ranges.

All 23 compounds were located in the HIA 99% confidence ellipse, and the absorption grade (Table 1) indicated that all the compounds had good absorption except one, which had moderate absorption. BBB grade predictions indicated that all compounds had medium or very low BBB permeability [24]. Regarding the CYP2D6 inhibition predictions, no compounds inhibited this enzyme and none cause serious drug interaction toxicity. As drug activity is related to free drug concentration, it is necessary to consider whether each compound may bind to plasma proteins [25]. The 23 candidate compounds all had weak plasma protein binding activity, with binding rates <90%. Regarding solubility predictions, 17 compounds had extremely high solubility, five had good solubility, and only one had low solubility. 

Next, molecular docking virtual screening (including LibDock and CDOCKER analyses, based on the structural matching degree analysis involving the target DPP-IV protein) were conducted for the 23 candidate compounds. The LibDock program involves a heatmap matching simulation based on the structures of immobilized molecules and the receptor protein [26]. LibDock calculates a heatmap for the active site of the receptor protein, which contains polar and nonpolar interaction sites, and then the ligands with various conformations are rigidly superimposed onto the map to determine the most suitable interaction and energy optimization [27]. For each compound, the conformation with the highest docking score can be obtained, and the compounds can then be listed by docking score. The binding site in the co-crystal structure (Protein Data Bank ID: 6B1E) of the drug vildagliptin (positive control) and the DPP-IV enzyme was selected to be the receptor binding site. The 23 candidate ligand compounds were subjected to the “prepare ligands” module to generate 352 configurations of ligands to match with the receptor. Seven out of the 23 molecules had higher LibDock scores than vildagliptin (Table 2). 

The seven compounds then underwent docking screening using the CDOCKER program. CDOCKER is a semi-flexible molecular docking analysis method based on the CHARMm force field, which can produce high-precision docking results, and it provides information on the interaction binding energy and ligand–receptor docking mode [28]. According to the molecular docking results of CDOCKER (Table 3), CDOCKER interactions and binding energies for three of the seven compounds were better than vildagliptin [29]. 

**Figure 2:**
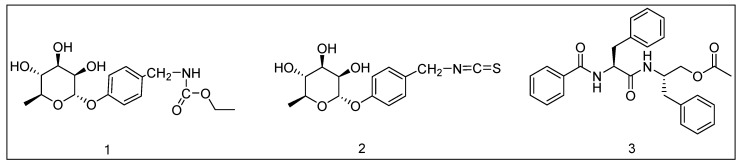
Molecular structure of compounds **1**–**3**.

Therefore, compounds **1**–**3** may be potential DPP-IV inhibitors based on the above virtual screening and docking processes. The molecular structures of the three compounds are shown in Figure 2.

**Figure 3:**
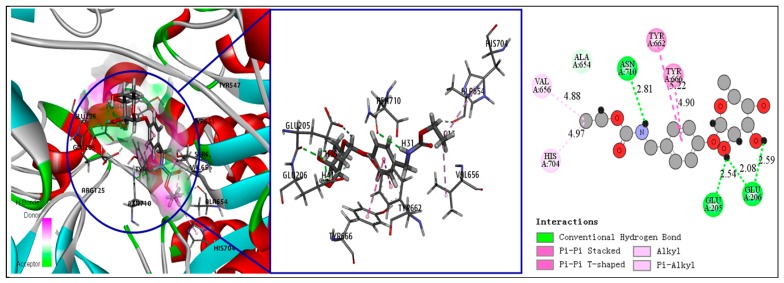
Receptor–ligand interaction of compound **1** with dipeptidyl peptidase (DPP-IV) binding site.

Understanding ligand–receptor interactions in depth provides a basis for the subsequent optimization of drug structures. We analyzed the docking modes of the three screened compounds based on the CDOCKER analysis. As a reference, vildagliptin and the three amino acid residues of the DPP-IV binding site each formed four hydrogen bonds, and vildagliptin’s adamantane fragment and tetrahydropyrrolidine fragment also formed three hydrophobic interactions with DPP-IV. Regarding the docking mode of compounds **1**–**3**, it was found that, like vildagliptin, each compound could form various hydrogen bonds and hydrophobic interactions with key amino acid residues at the DPP-IV binding site. The details are shown in Table 3. The compounds in the table are arranged according to the binding energy; the larger the value of the CDOCKER interaction energy and the lower the negative value of the binding energy (ΔG), the stronger the ligand–receptor interaction force [30]. Compound **1** is a unique urethane compound found in MO seeds. Each fragment of compound **1** can form various interaction bonds with DPP-IV (Figure 3). The N atom and glycosyl side chain of the urethane moiety and the O atom of the chain form four hydrogen bonds with the Asn710, Glu205, and Glu206 residues of DPP-IV, respectively, and the benzene ring can form two π–π interactions with the Tyr662 and Tyr666 residues. The ethane fragment of the side chain can also interact with the Val656 and His704 residues to form two alkyl hydrophobic forces. Faiz et al. reported that compound **1** has a certain hypotensive effect [31], but there are no reports on hypoglycemic activity or DPP-IV inhibition for compound **1**.

**Figure 4:**
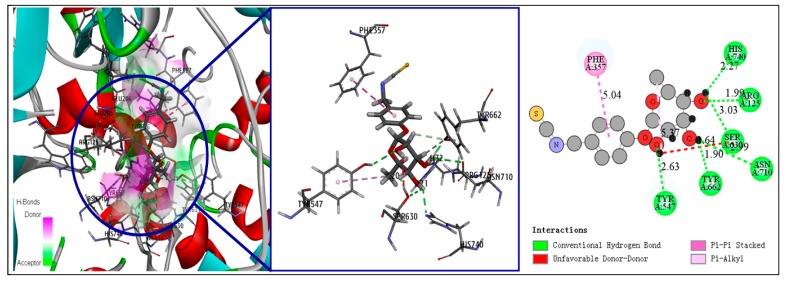
Receptor–ligand interaction of compound **2** with DPP-IV binding site.

Compound **2** is a major isothiocyanate active ingredient found in MO seeds. It can form six hydrogen bonds with DPP-IV, three O atoms, and two H atoms of the glycosyl side chain, and can interact with six residues in the DPP-IV binding site, at the His 740, Arg125, Ser630, Asn710, Tyr662, and Tyr547positions. The benzene ring of compound **2** can form a π–π stacked interaction with the Phe357 residue. However, it can be seen from Figure 4 that the H30 atom of compound **2** forms an unfavorable hydrogen bond with the Ser630 residue, and the key isothiocyanate group did not form any interaction with DPP-IV. This may have resulted in the lower binding energy for compound **2** compared to compound **1**. Carrie et al. reported that the addition of 5% compound **2**-rich MO extract to mouse feed can inhibit the rate-limiting step in liver gluconeogenesis, thereby directly or indirectly increasing insulin signaling and sensitivity [32]. This study indicates the reliability of our screening method, but no molecular mechanism underlying the effect of compound **2** against DM has been reported. Our findings will be helpful for related research.

**Figure 5:**
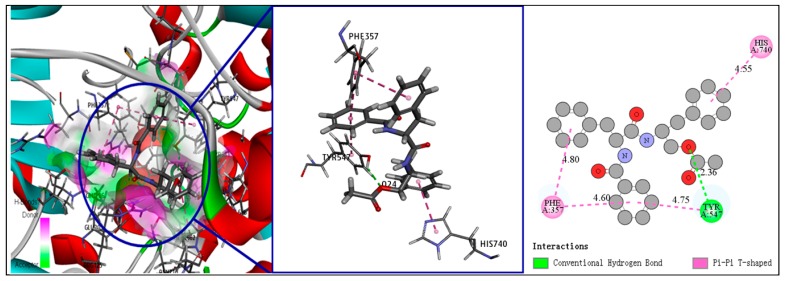
Receptor–ligand interaction of compound **3** with DPP-IV binding site.

Compound **3** is a dipeptide with a special structure that was first found in Aspergillus penicillioides. Isshiki et al. discovered that compound **3** relieves arthritis in rats by inhibiting Cathepsin L and B enzymes [33]. Yoon et al. found that compound **3** can inhibit the c-Jun N-terminal kinase (JNK) and p38 pathways to protect against nephritis induced by lipopolysaccharide (LPS) stimulation [34]. In our study, compound **3** and DPP-IV only formed one hydrogen bond, at the TYR (547 position (Figure 5)), while three benzene ring fragments formed four π–π interactions with the His704, Tyr547, and Phe357 residues. The number and types of interactions of compound **3** were fewer than those of compounds **1** and **2**. As a result, compound **3** had the lowest binding energy related to DPP-IV among the three compounds.

**Figure 6:**
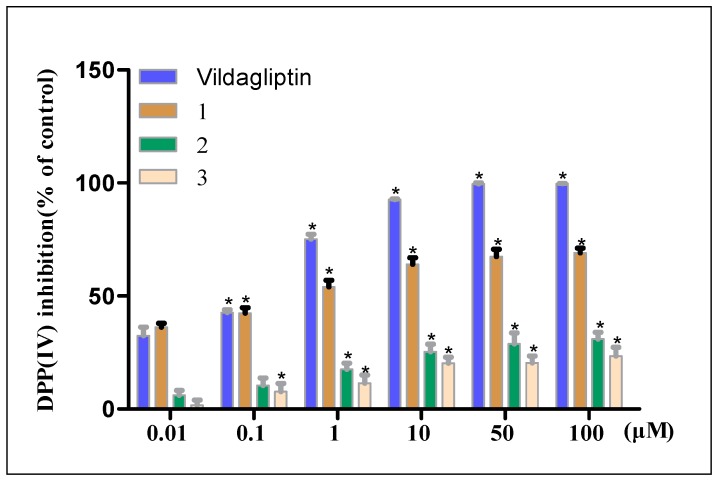
Inhibitory activity of compounds **1**–**3** and vildagliptin (at different doses) against DPP-IV. * *p* < 0.05 compared with the 0.01 μM group.

After the four-step virtual screening of 111 MO phytochemicals, we identified three potential DPP-IV inhibitors and purchased these compounds. Thereafter, in vitro fluorescence detection of inhibitory activity against DPP-IV was performed, and the half-maximal inhibitory concentration (IC_50_) values of compounds **1**–**3** were calculated. As shown in Figure 6, the three MO compounds inhibited the activity of DPP-IV to a certain extent, and the inhibitory activity was consistent with the order of the CDOCKER results. The inhibitory activity of compound **1** was the strongest among the three compounds, with an IC_50_ of 798 nM, which is equivalent to that of the positive control, vildagliptin (IC_50_ = 528 nM). The inhibitory activity of compound **1** increased with the concentration, showing considerable concentration dependence. When the compound concentration reached 100 μM, the inhibition rates were 99.64%, 71.25%, 30.93%, and 23.46% for vitagliptin and compounds **1**, **2**, and **3**, respectively. The IC_50_ values of compounds **2** and **3** were 157.694 μM and 191.126 μM, respectively, and both exhibited concentration dependence, but the DPP-IV inhibitory activity was moderate so no higher-concentration activity assay was performed.

In this study, for the first time, MO compound **1**, was found to be an excellent new type of DPP-IV inhibitor, making it a potential lead compound for the treatment of type 2 DM. The findings also suggest that the natural products’ binding ability and selectivity toward the protein target still need to be improved compared to those of commercially available drugs. In the future, a series of derivatives could be rationally designed and synthesized according to the ligand–receptor interaction mode results in order to improve the affinity of the compounds to the target.

Our research also led to detailed in silico predictions of the toxicological properties of compounds **1**–**3**. Table 4 shows the toxicological and chemical properties of the compounds. The numbers of hydrogen bond acceptors, hydrogen bond donors, ionization states, stereoisomers, and tautomers conformed to Lipinski’s “rule of five”, and the compounds exhibited good drug-like properties. Additionally, as shown in Table 1, compounds **1**–**3** had good ADME pharmacokinetic properties, and the prediction confidence was >95%. As shown in Table 4, compounds **1** and **3** (but not 2) were predicted to be biodegradable. All compounds were predicted to be noncarcinogenic for male and female rats and mice according to the US National Toxicology Program (NTP) classification regarding evidence of carcinogenic activity. Similarly, all compounds had high safety regarding predicted carcinogenicity according to the Ames mutagenicity and weight of evidence (WOE) results [35]. Compounds **1** and **2** had predicted hepatotoxicity, so improvements need to be made through further structural optimization. The skin sensitization evaluation indicated that all compounds are mild and nonirritating to the skin. Regarding the prediction of the median toxic dose (TD_50_) and median lethal concentration (LC_50_), compound **1** had a higher safe dose than vildagliptin, indicating that compound **1** is a good potential DPP-IV inhibitor.

## 3. Materials and Methods 

### 3.1. Literature Search and Establishment of Ligand Library

PubMed and Google Scholar were used to identify reports published before December 2019 on the isolation of phytochemicals of MO. The 2D structures were downloaded from the PubChem database by name, or drawn using ChemDraw software. Discovery Studio 4.5 software (Accelrys Software Inc., San Diego, CA, USA) was used to convert each 2D structure to a 3D molecular formula, and the “Prepare Ligands” module was used to add hydrogen atoms and perform energy optimization operations. 

### 3.2. First-Round Screening Using Lipinski’s “Rule of Five”

The “filter molecule” module in Discovery Studio was used, with parameters selected based on Lipinski’s “rule of five.” The molecular structures in the MO compound library that did not conform with Lipinski’s “rule of five” (regarding conventional drug properties) were discarded. According to Lipinski’s “rule of five”, a reasonable candidate for use as an orally active drug should have no more than one violation of the following criteria: <5 hydrogen bond donors, <10 hydrogen bond acceptors, molecular weight < 500, AlogP < 5, and no more than one violation of the above criteria. After this, the “prepare ligands” module was applied to the remaining molecules to generate multiple conformations.

### 3.3. Second-Round Screening on the Basis of ADME/T Properties

The “ADME/T Descriptor” module of DS was used. In the parameter settings, water solubility, BBB penetration, CYP2D6 binding, liver toxicity, intestinal absorption, and plasma protein binding were selected as the research objects. In addition, more detailed toxicity predictions were performed for compounds **1**–**3**. In the parameter settings of the “toxicity prediction” module, carcinogenicity, mutagenicity, skin irritation, TD_50_, and LC_50_ were selected as the research objects. Compounds that had poor pharmacokinetic properties and were likely to have high carcinogenic and mutagenic potential were excluded.

### 3.4. Third-Round Screening Using LibDock 

Before LibDock screening, it was necessary to determine the binding site. Thus, the vildagliptin-binding site in the X-ray crystal structure of DPP-IV in complex with vildagliptin (Protein Data Bank ID: 6B1E) was determined by co-crystallization (X: 35.8402, Y: 50.2541, and Z: 35.3156), and the radius was set to 12 Å. The ligand conformation generation method was selected as “best”, the number of binding site hotspots was set to 100, and other parameters were set at their default values. The docking results were sorted by LibDock score.

### 3.5. Fourth-Round Screening Using CDOCKER Molecular Docking Analysis and Docking Mode Analysis

For the molecular docking analysis, the crystal structure of DPP-IV in complex with vildagliptin (Protein Data Bank ID: 6B1E) was selected as the acceptor, and it was optimized by hydrogenation and CHARMm force field calculations. The binding site was defined by the ligand atoms, and the radius range was automatically generated. The CHARMm force field and annealing simulation algorithm were used to optimize the energy of the complexes of ligands with the protein, combining them in different conformations. Parameters were set at their default values. After each compound was docked, the 10 best conformations were obtained. The compounds were screened by comprehensively considering their interaction energy and binding free energy. The analysis of the binding mode (3D or 2D ligand–receptor interaction simulation map) of each selected compound was also conducted using CDOCKER. 

### 3.6. In Vitro DPP-IV Inhibition Assay 

A DPP-IV Inhibitor Screening Assay Kit (KA1311) was purchased from Abnova Co. Ltd. (Taipei, Taiwan). Compounds **1**–**3** and the positive control drug vildagliptin were purchased from BioBioPha Co., Ltd. (Kunming, China). The in vitro inhibitory activity of each compound against DPP-IV was determined based on a fluorescence detection method. In brief, 10 μL of different concentrations of each compound (0.01, 0.1, 1, 10, 50, and 100 μM) were mixed with 30 μL of DPP assay buffer and 10 μL of DPP-IV, giving a total reaction volume a 50 μL. Additionally, a “100% initial activity well” (10 μL dimethyl sulfoxide [DMSO], 30 μL DPP assay buffer, and 10 μL DPP-IV) and a “background well” (10 μL DMSO and 40 μL DPP assay buffer) were prepared. Next, 50 μL of DPP was added to each well and incubated at 37 °C for 30 min. A FlexStation 3 multifunctional microplate reader (Molecular Devices, Sunnyvale, CA, USA) was used to detect the fluorescence value (excitation wavelength: 350–360 nm; emission wavelength: 450–465 nm). The inhibition rate of each sample was calculated as follows: inhibition rate% = [(100% initial activity well-background well) − (sample well-background well)]/(100% initial activity well-background well) × 100%. IC_50_ values were then calculated using GraphPad Prism 5 (GraphPad Software, La Jolla, CA, USA).

## 4. Conclusions

To identify the potentially anti-diabetic active components of MO, we carried out computer-assisted virtual screening of phytochemicals from MO based on the structure of the DPP-IV enzyme, and we verified the candidate compounds using an in vitro DPP-IV inhibition assay. For the first time, three natural MO components with inhibitory activity against DPP-IV were identified. Among them, the most effective compound was compound **1** (IC_50_ = 798 nM), which is a urethane known as O-Ethyl-4-[(α-l-rhamnosyloxy) benzyl] carbamate. It has excellent pharmacokinetic properties and safety and is a potential lead compound against DPP-IV. Additionally, a molecular docking analysis was used to simulate the interaction mode of the candidate compounds with the DPP-IV receptor, which provides the necessary basis for subsequent structural optimization and drug research.

## Figures and Tables

**Table 1:** Chemical names and pharmacokinetic parameters of 23 compounds selected after absorption, distribution, metabolism, excretion, and toxicity (ADME/T) screening.

Compound Number	Absorption Level ^a^	BBB Level ^b^	CYP2D6 Inhibition	Plasma Protein Binding	Solubility Level ^c^
**1**	0	4	No	Weak	4
**2**	0	3	No	Weak	4
**3**	0	2	No	Weak	2
**4**	0	4	No	Weak	4
**5**	0	4	No	Weak	4
**6**	0	3	No	Weak	3
**7**	0	3	No	Weak	4
**8**	0	4	No	Weak	4
**9**	0	2	No	Weak	4
**10**	0	2	No	Weak	4
**11**	0	3	No	Weak	3
**12**	0	4	No	Weak	3
**13**	0	3	No	Weak	3
**14**	0	3	No	Weak	4
**15**	0	3	No	No	4
**16**	0	3	No	No	4
**17**	0	3	No	No	4
**18**	0	3	No	No	4
**19**	0	3	No	No	4
**20**	1	4	No	No	4
**21**	0	2	No	No	4
**22**	0	3	No	No	3
**23**	0	3	No	No	4

a 0: good absorption; 1: moderate absorption. b 2: medium penetration; 3: low penetration; 4: undefined; c 2: low solubility; 3: good solubility; 4: optimal solubility. BBB: blood–brain barrier; CYP2D6: cytochrome P450 2D6.

**Table 2:** LibDock scores for compounds **1**–**7** and vildagliptin.

Compound Number	LibDock Score
**3**	data
**5**	120.126
**1**	110.991
**6**	109.801
**4**	103.673
**7**	102.232
**2**	99.719
Vildagliptin	93.424

**Table 3:** CDOCKER results and docking mode analysis results for compounds **1**–**3** and vildagliptin.

Compound Number	CDOCKER Interaction Energy (kcal/mol)	Binding Energy (kcal/mol)	Number of Hydrogen Bonds	Number of Hydrophilic Bonds
**1**	44.9575	−84.9987	4	4
**2**	39.3594	−81.1002	6	1
**3**	35.7187	−47.3644	1	4
Vildagliptin	35.6244	−42.0109	4	3

**Table 4:** Chemical information and toxicity properties of compounds **1**–**3** and vildagliptin.

Compound	1	2	3	Vildagliptin
Molecular weight	341.36	311.35	377.39	303.40
H-bond acceptor	8	6	8	5
H-bond donor	4	3	4	2
No. of ionization states	1	1	1	3
No. of tautomers	1	1	1	1
Aerobic biodegradability	Degradable	Nondegradable	Degradable	Degradable
Ames mutagenicity	Nonmutagen	Nonmutagen	Nonmutagen	Nonmutagen
Mouse NTP classification ^a^	Noncarcinogen	Noncarcinogen	Noncarcinogen	Noncarcinogen
Rat NTP classification ^a^	Noncarcinogen	Noncarcinogen	Noncarcinogen	Noncarcinogen
WOE prediction	Noncarcinogen	Noncarcinogen	Noncarcinogen	Noncarcinogen
Hepatotoxicity	Yes	Yes	No	No
Skin sensitization	Mild	Mild	Mild	Mild
TD50 (mg/kg)	32.81	19.78	5.14	1.48
LC50 (g/L)	0.58	0.15	0.05	0.26

LC_50_: median lethal concentration; NTP: national toxicology program; TD_50_: median toxic dose; WOE: weight of evidence; ^a^ male and female model.

## Supplementary Materials

The following is available online. Table S1: Summary of the 111 chemical constituents of *Moringa oleifera* (MO) used in the virtual screening in this study.
